# A comparative dataset on the GC–MS analysis and antioxidant activity of *Selaginella bryopteris* from different geolocations of India

**DOI:** 10.1016/j.dib.2025.111318

**Published:** 2025-01-22

**Authors:** Utkarsha Srivastava, Ashwani Mathur

**Affiliations:** Department of Biotechnology, A-10 Jaypee Institute of Information Technology, Sector-62, Noida 201310, India

**Keywords:** *Selaginella bryopteris*, GC–MS, Alcoholic extraction, DPPH, FRAP, TAC

## Abstract

*Selaginella bryopteris* (L.) Baker, is a lithophytic pteridophyte of the Selaginellaceae family. It has been reported for its medicinal properties due to its rich phytocompound pool. The current work reveals a comprehensive dataset on the phytochemical profile of this species procured from different geolocations in the deccan terrain of India. The extraction was undertaken with four solvents namely, water, ethanol, methanol and acetone. The phytocompound analysis was done using qualitative detection methods. The acetone and methanolic extracts were analysed for their phytochemical composition, using GC–MS (Agilent 5977B EI/CI MSD), on the basis of area (%) covered in the chromatogram up to the retention time (RT) of 23 min. The GC–MS analysis of plant from Tamil Nadu showed the maximum number of peaks in both the acetone and methanolic extracts (83 and 74 respectively). A total of 12 and 14 peaks were observed in the chromatogram of methanolic extracts of plant from Karnataka (SBK) and Maharashtra (SBM), respectively. The acetone extracts of Karnataka and Maharashtra showed 77 and 42 peaks respectively. Some common compounds tentatively identified by GC–MS analysis were Hexadecanoic acid, methyl ester, trans-13-Octadecenoic acid, methyl ester and Eugenol. The comparative antioxidant assessment of the extracts from all three geolocations was undertaken by three different methods of free radical scavenging activity by DPPH assay, FRAP assay and total antioxidant capacity determination by Phosphomolybdate assay. Ethanolic and methanolic extracts showed nearly similar IC_50_ for all the antioxidant assays. The results were statistically evaluated using Data Analysis tools of Microsoft Excel. Single factor ANOVA (*p* < 0.05) was used to determine the significance of data. The present results add to the limited data available on the comparative analysis of antioxidant potential and GC–MS phytochemical profile of *Selaginella bryopteris*.

Specifications Table

**Table:** 

Subject	Biological Sciences
Specific subject area	Medicinal plants and pharmacology
Type of data	Chromatogram images, graphs, figures, tables, raw
Data collection	*The aqueous and alcoholic extracts of raw plant material of S. bryopteris, procured from states of Karnataka, Maharashtra and Tamil Nadu (in India) were estimated for phytocompounds pool using method reported previously, with modification. The GC–MS analysis was done using Agilent 5977B EI/CI MSD and data analysed using NIST, Wiley and FAME databases for compounds based on area (%) up to RT of 23* min*. The data beyond 23* min *was excluded due to low area (%). The anti-oxidant analysis was performed using previously reported procedures. The colorimetric change for antioxidant assay was evaluated by spectrophotometric analysis using Jenway 6850 UV/vis spectrophotometer at different wavelengths (517 nm for DPPH assay, 593 nm for FRAP assay and 695 for TAC assay)*
Data source location	The plant material of *Selaginella bryopteris* was procured from three different geolocations of the deccan terrain of India, namely Chennai in Tamil Nadu (13°04′N latitude and 80°17′E longitude), Bengaluru in Karnataka (12.9716° N latitude and 77.5946° E longitude) and Kolhapur in Maharashtra (16.7050° N latitude and 74.2433° E longitude).
Data accessibility	Raw data has been submitted to and is accessible at Mendeley Data repository with the DOI: 10.17632/hppt3xj39r.10.17632/hppt3xj39r.2 Direct link to the data: https://data.mendeley.com/datasets/hppt3xj39r/2

## 1 Value of the Data

• Traditionally known as “*Sanjeevani”, Selaginella bryopteris* is a valuable pteridophyte herb, well documented in ancient literature and folklore. The species is endemic to India and the Indian subcontinent and may be found in countries of Nepal and Sri Lanka. It is widely used for alleviating neurological conditions and disease modulation. It grows best under the shades of tall plants, where it receives filtered light, in regions with moderate temperature preferably ranging from 15 °C to 25 °C. Humidity and water content dominantly regulate its growth and proliferation. Areas with high humidity and preferably running water as the streams and waterfall are best suited for the growth of this plant. The fronds of this species curl under unfavorable conditions which are revived to their normal physiological conditions upon the return of favorable environment. The resurrection properties of this herb make it a suitable choice for growth in limited water conditions although the availability of water and the variations in environmental conditions define the phytocompound pool of the species.
• Variations in the demography play a significant role in catalogues of phytocompounds of *Selaginella bryopteris*, and associated therapeutic activities. The physiological factors such as climate, soil composition, water content and altitude profoundly influence the metabolites of the plant. The effect of these variations can be observed both morphologically and in the phytochemical composition of the species. Several studies have reported such differences in composition for angiosperms and gymnosperms, but such data on pteridophytes is limited making this evaluation extremely crucial. The remarkable resilience of pteridophytes to these variations have a huge impact in defining the phytocompound pool. This makes it important to assess the therapeutic composition of these plants with respect to the changing conditions which alter the composition and the nature of the phytocompounds.
• *Selaginella bryopteris* harbors a distinctive profile of phytocompounds. The species has been reported for its signature compounds including selaginellin, amentoflavone and their derivatives most of which belong to the flavonoid group and exhibit anti-oxidant, anti-inflammatory, anticancer and neuroprotective properties. Further analysis of the secondary metabolites which may differ depending on the physiological conditions, can be evaluated by different techniques as GC–MS and LC–MS.
• The current work was undertaken to evaluate the effect of environmental conditions in regulating the phytocompound composition of *Selaginella bryopteris*. The plant, procured from different geolocations in India, showed morphological variations. Plants from the state of Maharashtra showed hard woody stem with sturdy fronds and small roots while that from Karnataka and Tamil Nadu had soft stem, longer roots and more branching and fronds. These differences motivated the present work which focuses on the phytocompound profile, as seen in the chromatograms results. The current study will assist the analysis of demography mediated changes in phytochemical profile, potential therapeutic benefits associated with phytocompounds and exploration of the conditions most suitable for the growth and propagation of the plants. The study will further pave the way for isolation of therapeutic phytocompounds, tentatively identified in the present study.

## 2 Background

*Selaginella bryopteris* (L.) Bak, is a lithophytic pteridophyte endemic to Indian sub-continent and found in the hilly terrains of India. Traditionally it is known as “Sanjeevani” in the ancient Indian scripture, “Ramayana” written by Maharishi Valmiki. It is also known as resurrection herb due to its drought resistant ability. The fronds of the plant curl during dehydrating conditions to prevent water loss which appear to revive when favourable conditions are restored [1,2]. *Charaka Samhita* and *Sushruta* have mentioned the medicinal properties of *S.bryopteris*. It is also used as a traditional Chinese medicine.

Other species of this genus have been explored extensively for their therapeutic properties. Although, the effect of habitat on the secondary metabolites of plants has been evaluated in multiple studies, the specifics for the current work help in further establishing the variations in phytocompound pool from the same species procured from overlapping geographical conditions. An understanding of these conditions will prove crucial in determining the same for the propagation of this plant for therapeutic purposes. The present work, was therefore, focused on evaluating the impact of the narrow range of environmental conditions on the phytocompound consortia. The effect of this small niche of environmental variations was morphologically visible in the plants, with the plants of Maharashtra having long woody stem with sturdy fronds and short fragile roots, while that of Karnataka and Tamil Nadu had more delicate stem and fronds with a more extensive root system.

## 3 Data Description

The present dataset is a representation of the variations in the phytochemical profile and GC–MS analysis of the different extracts of *Selaginella bryopteris*. The plant was procured from different geolocations in India, namely Bengaluru in Karnataka (referred to as SBK), Kolhapur in Maharashtra (referred to as SBM) and Chennai in Tamil Nadu (referred to as SBT). Table 1 gives an overview of the difference in the geographical locations of the three cities in India. It also gives an overview of the precipitation and average temperature range of these locations in the monsoon month of August, which is the most suitable for the propagation of this plant.

**Table 1:** Geographical location of the sites of plant procurement.

Location of procurement	Latitude	Longitude	Elevation (m/feet)	Temperature (in August)	Precipitation (in August)
Kolhapur (Maharashtra)	74.2433° E	16.7050° N	569 m/1867 feet	22–27 °C	280 mm
Bangaluru (Karnataka)	77.5946° E	12.9716° N	900 m/3000 feet	20–28 °C	122 mm
Chennai (Tamil Nadu)	80.2707° E	13.0827° N	6.7 m/22 feet	25–35 °C	137 mm

**Fig 1:**
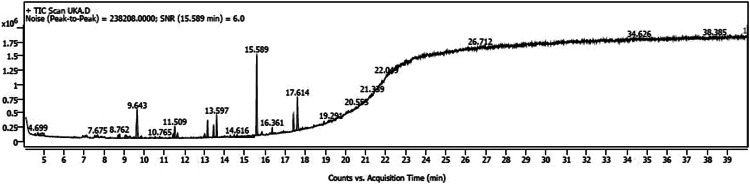
GC–MS chromatogram for acetone extract of *S.bryopteris* from Karnataka (SBK-A).

**Fig 2:**
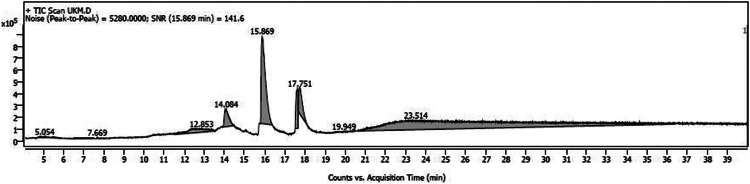
GC–MS chromatogram for methanol extract of *S.bryopteris* from Karnataka (SBK-M).

**Fig 3:**
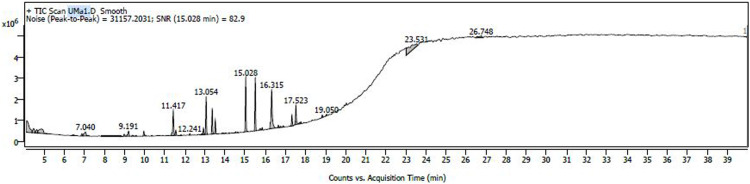
GC–MS chromatogram for acetone extract of *S.bryopteris* from Maharashtra (SBM-A).

**Fig 4:**
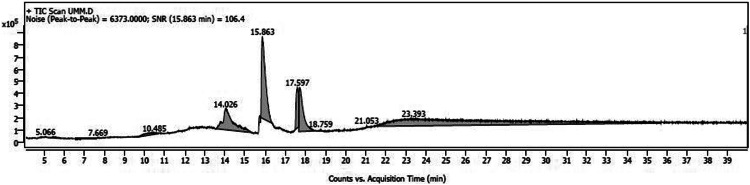
GC–MS chromatogram for methanol extract of *S.bryopteris* from Maharashtra (SBM-M).

**Fig 5:**
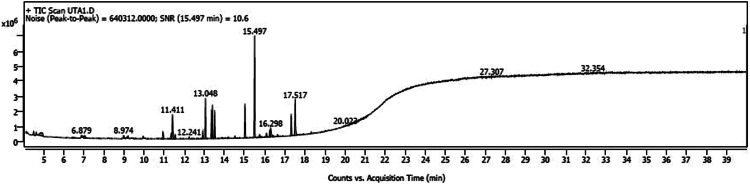
GC–MS chromatogram for acetone extract of *S.bryopteris* from Tamil Nadu (SBT-A).

**Fig 6:**
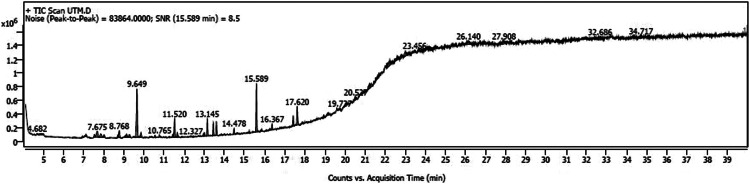
GC–MS chromatogram for methanol extract of *S.bryopteris* from Tamil Nadu (SBT-M).

The variations in the extractive yield of the aqueous and alcoholic solvent extracts of this plant is mentioned in Table 2. Fig 1, Fig 2, Fig 3, Fig 4, Fig 5, Fig 6 depicts the GC–MS chromatogram of the acetone and methanol extracts of the different samples. Additionally, the Table 3, Table 4, Table 5 give a comprehensive dataset of the probable compounds identified via GC–MS analysis of these extracts. The raw data of the GC–MS analysis is available in the Mendeley data repository by the DOI: 10.17632/hppt3xj39r.2

**Table 2:** Extractive Yield (in percentage) for the different samples of plant extracts^1^.

Extraction solvent	SBK	SBM	SBT
Water	4.09	4.44	2.67
Methanol	2.34	8.13	6.1
Ethanol	1.9	6.1	5.61
Acetone	1.24	3.29	1.06

**Table 3:** Provisionally identified phytochemicals from the acetone and methanol extracts of Karnataka (SBK) based on the percentage of area in the GC–MS chromatogram.

S.No	RT (min)	NIST DATABASE/Wiley 2007/FAME ID/ (acetone)	Area (%)		S.No	RT (min)	NIST DATABASE/Wiley 2007/FAME ID/ (methanol)	Area (%)
1.	9.643	2-Isopropyl-5-methylcyclohexyl methylphosphonofluoridate	57.08		1.	15.869	Hexadecanoic acid, methyl ester	28.72
2.	17.614	13-Octadecenoic acid, methyl ester	49.88		2.	17.751	trans-13-Octadecenoic acid, methyl ester	7.52
3.	13.597	12-methyl-Tridecanoic acid, methyl ester	26.05		3.	12.853	1,4-Methanocycloocta[d]pyridazine, 1,4,4a,5,6,9,10,10a-octahydro-1,11-dimethyl-, (1.alpha.,4.alpha.,4a.alpha.,10a.alpha.)-	6.35
4.	13.145	trans-2-Methyl-4-n-butylthiane, S,S-dioxide	21.54		4.	7.669	Glycopyrrolate	0.08
5.	17.42	16-methyl-Heptadecanoic acid, methyl ester	20.99

**Table 4:** Provisionally identified phytochemicals from the acetone and methanol extracts of Maharashtra (SBM) based on the percentage of area in the GC–MS chromatogram.

S No.	RT (min)	NIST DATABASE/Wiley 2007/FAME ID/ (acetone)	Area (%)		S No.	RT (min)	NIST DATABASE/Wiley 2007/FAME ID/ (methanol)	Area (%)
1.	15.028	Eugenol	84.16		1.	15.863	Hexadecanoic acid, methyl ester	38.40
2.	15.503	Hexadecanoic acid, methyl ester	79.28		2.	14.026	4,5,6,7-Tetrahydroindazole-3-spirocyclohexane	25.19
3.	11.417	1-isocyanato-3-methoxy- Benzene	47.99		3.	17.734	trans-13-Octadecenoic acid, methyl ester	22.50
4.	13.511	12-methyl- Tridecanoic acid, methyl ester	19.98		4.	17.597	1,3-butadienylidene Cyclohexane	11.23
5.	7.04	(2S,13S)−12,13-Dihydroxy-1,4,7,10-tetraoxacyclotetradecane	17.42		5.	10.485	n-Dodecylpyridinium chloride	2.84

**Table 5:** Provisionally identified phytochemicals from the acetone and methanol extracts of Tamil Nadu (SBT) based on the percentage of area in the GC–MS chromatogram.

S.No	RT (min)	NIST DATABASE/Wiley 2007/FAME ID/ (acetone)	Area (%)		S No.	RT (min)	NIST DATABASE/Wiley 2007/FAME ID/ (methanol)	Area (%)
1.	17.517	Methyl 12,13-tetradecadienoate	44.89		1.	15.589	Hexadecanoic acid, methyl ester	70.58
2.	13.397	Phosphonic acid, (p-hydroxyphenyl)-	37.99		2.	7.675	L-Menthone	18.98
3.	15.016	Eugenol	36.04		3.	13.597	Tridecanoic acid, 12-methyl-, methyl ester	18.29
4.	11.411	Benzene, 1-isocyanato-3-methoxy-	33.48		4.	26.140	2-[2-[2-[2-[2-[2-[2-[2-[2-[2-(2- Methoxyethoxy)ethoxy]ethoxy]ethoxy]ethoxy]ethoxy]ethoxy]ethox y]ethoxy]ethoxy]ethanol	17.92
5.	13.511	Tridecanoic acid, 12-methyl-, methyl ester	26.24		5.	8.768	2-Isopropyl-5-methylcyclohexyl methylphosphonofluoridate	15.42
6.	17.322	1-Hexadecanaminium, N,N,N-trimethyl-, octadecanoate	21.20		6.	7.847	Benzofuran, 4,5,6,7-tetrahydro-3,6-dimethyl-	10.78
7.	16.298	2-(diethylboryloxy)- Ethanethiol	16.16					
8.	11.537	6,6-Dimethyl-2-vinylidenebicyclo[3.1.1]heptane	6.12					

**Fig 7:**
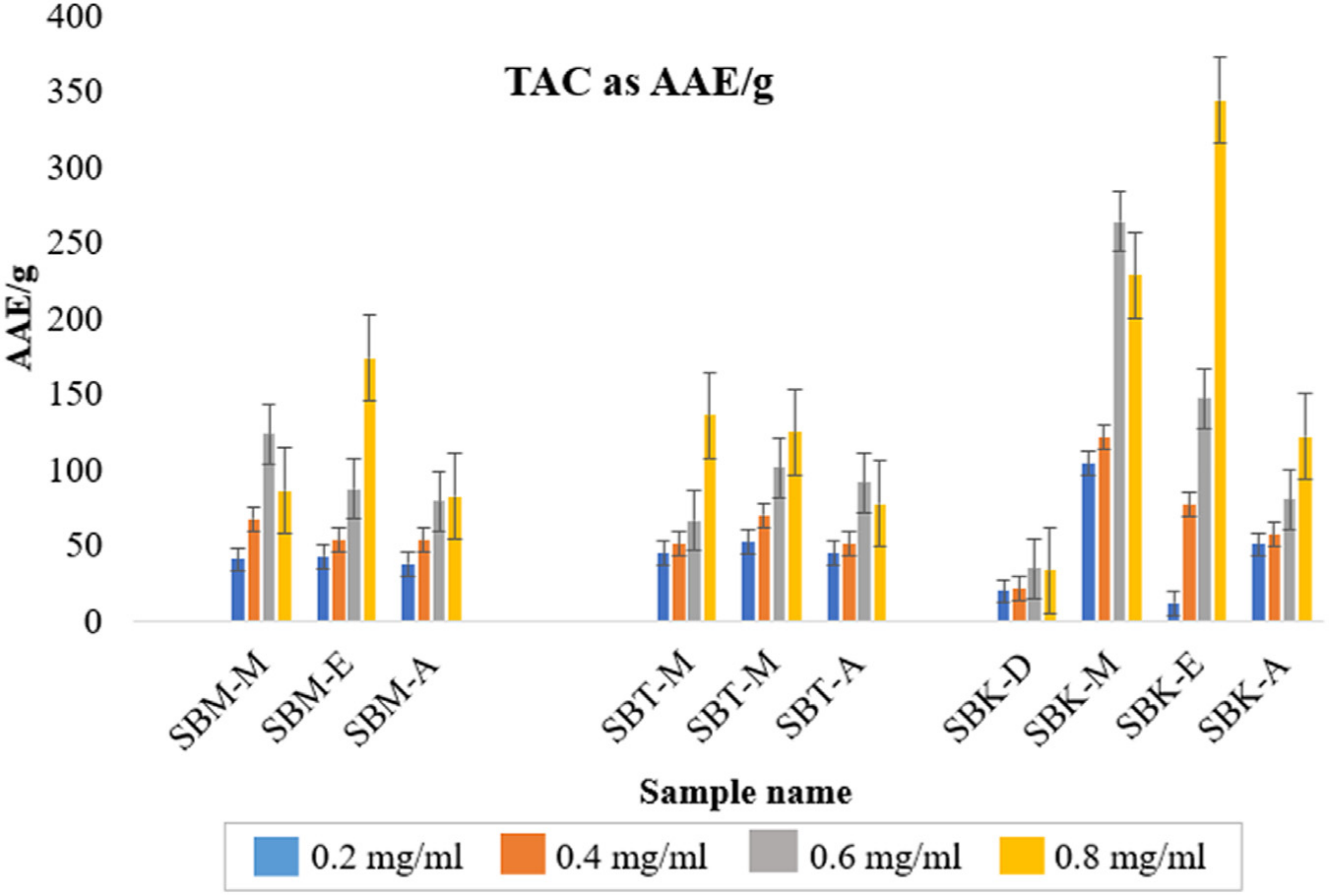
Phosphomolybdate assay (Total antioxidant capacity) for various concentrations of plant extracts^2^.

**Fig. 8:**
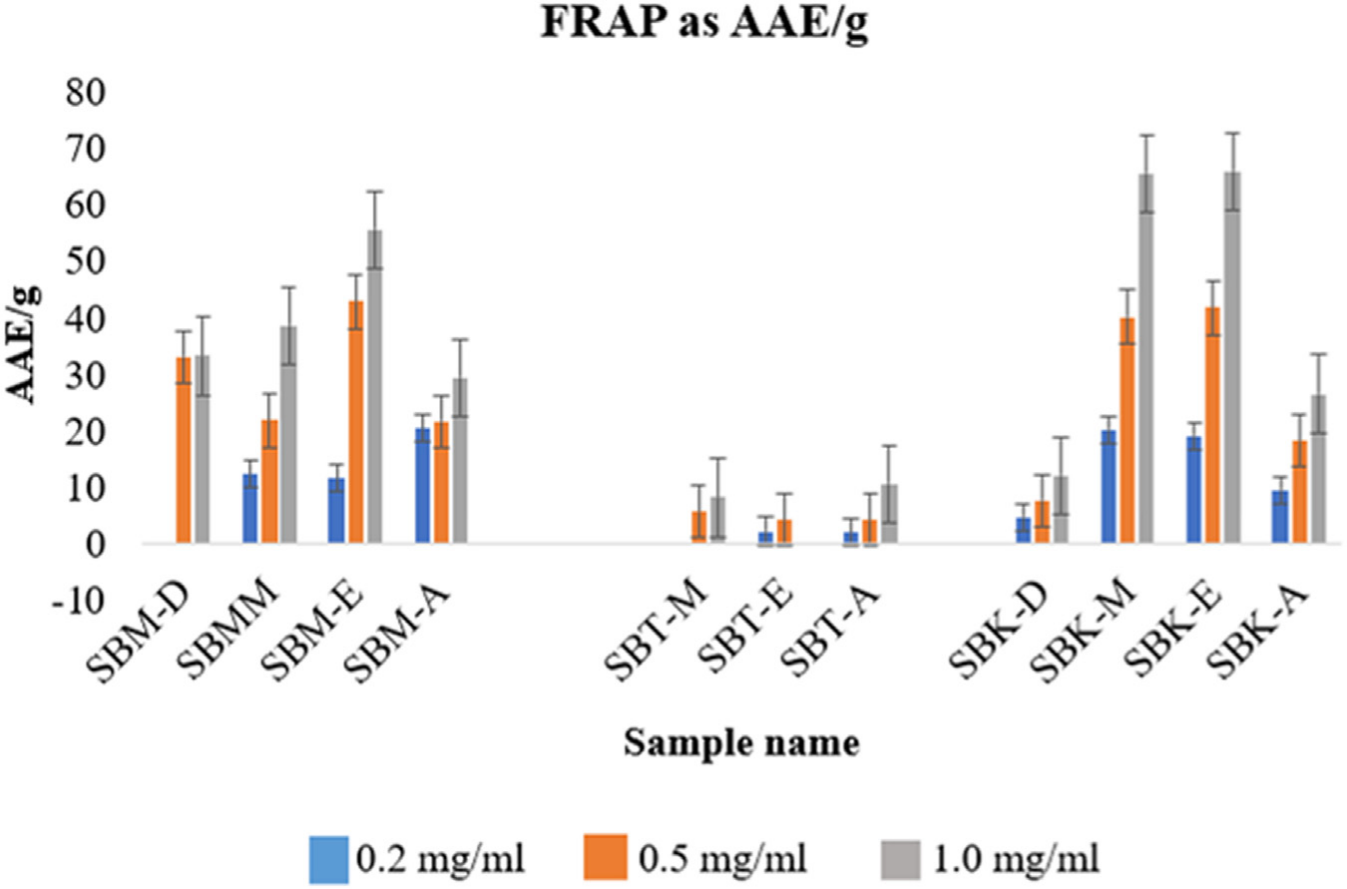
FRAP assay at four different concentrations of extracts.

The antioxidant capacity assays for all the extracts, were performed using three different procedures viz. DPPH free radical scavenging activity, Total Antioxidant Capacity and FRAP assay (Fig 7, Fig. 8). Table 6 enlists the concentration of these extracts required for 50 % inhibition of the assay radicals as per the concentration of the assay compound used. It is reported as inhibition concentration (IC_50_) of the samples as antioxidant.

**Table 6:** IC_50_ of test samples by different antioxidant activity assessment methods (ND means Not Determined).

Sample name	IC50 for DPPH (µg/ml)	IC50 for Phosphomolybdate Assay (µg/ml)	IC50 for FRAP Assay (µg/ml)
**SBT-D**	0.40	ND	ND
**SBT-M**	0.56	0.50	0.54
**SBT-E**	0.56	0.52	0.75
**SBT-A**	0.55	0.54	0.55
			
**SBK-D**	0.41	0.55	0.62
**SBK-M**	0.47	0.52	0.60
**SBK-E**	0.50	0.44	0.60
**SBK-A**	0.73	0.52	0.62
			
**SBM-D**	0.39	ND	0.58
**SBM-M**	0.53	0.70	0.60
**SBM-E**	0.53	0.48	0.54
**SBM-A**	0.53	1.75	0.69

## 4 Experimental Design, Materials and Methods

### 4.1 Collection of plant material and preparation of plant extract

The plant material was procured from three different geolocations in India, namely, Kolhapur district of Maharashtra, district Bengaluru of Karnataka and Chennai district of Tamil Nadu (Table 1). The plants from Karnataka (SBK) and Tamil Nadu (SBT) were received as potted plants while the plant from Maharashtra (SBM) was received as dry material which was revived by keeping the plant soaked in water for 24 h. The samples were identified at the Department of Forest Products, Dr. Y.S. Parmar University of Horticulture and Forestry, Solan, Himachal Pradesh, India.

The dried sample was washed with distilled water and coarsely grounded using mortar and pestle. Plant extracts were prepared as previously mentioned by Sarvade et al [3] with slight modifications. Briefly, 15 g grounded sample was extracted by cold maceration technique in 100 ml of different solvents, water, ethanol, methanol and acetone. The extraction was performed with agitation at 120 rpm and 37 °C. Extracts were filtered using Whatman filter paper 1 and the filtrate was dried and weighed. The percent extractive yield has been mentioned in Table 2. The plant material from Kolhapur, Bengaluru and Chennai have been coded SBM, SBK and SBT respectively. The dried extract was stored at −20 °C until further use. The dried extracts were reconstituted in methanol for assessment of antioxidant activity.

### 4.2 Gas chromatography and mass spectroscopy (GC–MS) analysis

The methanolic and acetone extracts of the plant samples (without derivatization) were subjected to Gas chromatography - Mass Spectroscopy (GC–MS) analysis. The GC–MS instrument was from Agilent 5977B EI/CI MSD, with a mass range of 30–550 amu. The column oven temperature was maintained at 80 °C with a hold time of 2 min. The column flow rate was held at 1.21 ml/min at a temperature of 280 °C and a hold time of 18 min. The time for the start and end of MS program was 4–40 min at a scan speed of 3333. The analysis of the unknown components was done in comparison with the data available from the “NIST MS Database”, “Wiley GC–MS library 2007″ and FAME.

### 4.3 Assessment of antioxidant capacity of extracts

#### 4.3.1 DPPH assay

DPPH is chemically known as ɑ, α-diphenyl-β-picrylhydrazyl. It was used to determine the antioxidant capacity of the plant extracts reconstituted in methanol. DPPH (3 mM) solution was prepared in methanol. Different concentrations of Ascorbic acid, used as standard, were prepared from 50 µg/ml to 250 µg/ml in methanol. Test sample was mixed with DPPH solution in the ratio of 3:1 and incubated for 30 min in dark. The absorbance was taken at 517 nm using Jenway 6850 UV/vis spectrophotometer. The percent inhibition of free radical formation is indication of the antioxidant capacity of the sample and was calculated using the formula mentioned below [4,5]

$$\text{Percent}\;\text{Inhibition}=1-\frac{Asample}{Acontrol}\times \;100$$

where A _sample_ is the absorbance of the sample at 517 nm and

A _control_ is the absorbance of the control solution (Ascorbic acid) at 517 nm

Table 6 shows the IC_50_ of these extracts for their free radical scavenging activity.

#### 4.3.2 Total anti-oxidant capacity (TAC) using phosphomolybdate assay

Total antioxidant capacity of the sample was measured by the phosphomolybdate assay which works on the principle of reduction of Mo (VI) to Mo(V) by the antioxidant compounds. Ascorbic acid was used as standard at a concentration of 1 mg/ml. Reaction mixture consisted of ammonium molybdate (0.4 M), sulfuric acid (0.6 M), sodium phosphate (0.25 M) and sample. This reaction mixture was incubated at 95 °C for 1 h 30 min and left to cool. Absorbance of the mixture was taken at 695nm. The total antioxidant capacity was measured as Ascorbic acid equivalent [6,7]. The results have been shown in Fig 7 and the IC_50_ value is reported in Table 6.

#### 4.3.3 Ferric reducing antioxidant power assay (FRAP)

Ferric reducing antioxidant power (FRAP) works on the principle of reduction of Fe^3+^ to Fe^2+^ from its tripyridyltriazine solution by the action of antioxidants. Ferric chloride solution (20mM) was added to acetate buffer (pH 3.6) in the ratio of 1:10. The solution, 2,4,6-Tris(2-pyridyl)-s-triazine (TPTZ) (40mM) was added to this mixture in the same ratio. The FRAP reagent was warmed at 37 °C for 10 min before use. Freshly prepared FRAP reagent was used for analysis. FRAP reagent and sample were mixed in the ratio of 20:1 and incubated in dark for 30 min. The absorbance was taken at 593 nm [6]. Ascorbic acid was used as standard. The IC_50_ of the extracts for total antioxidant capacity has been reported in Table 6 while Fig. 8 gives an the AAE/g of the extract at four different concentrations of the extracts.

### 4.4 Statistical analysis

All the results were verified statistically using Data Analysis tool of Microsoft Excel (Version 2310). All the studies were performed in triplicates (sample). The linear regression coefficient (R^2^) for all the studies was evaluated using Microsoft Excel. Single factor ANOVA with a *p*-value of less than 0.05 was used to test the significance of data. Student's *t*-test was performed for all the samples. The results were statistically significant.

## Limitations

The age of plant. time and season of collection of the plant material will play a significant role in determining its phytochemical profile since the environmental factors play an important role in governing the production of secondary metabolites in plants. As the authors depend upon the procurement of plants from registered vendors (within India), limited information has been provided about age, time and season of harvest. The presence of silicone rich compounds may lead to ambiguities during analysis of phytochemical profile by GC–MS. The variations in phytocompounds, yield and their analysis may differ from plant of one habitat to another.

## Ethics Statement

The authors confirm that they have read and followed the ethical requirements for publication in Data in Brief. The authors further confirm that the current work does not involve animal experiments, human subjects or any data collected from social media platforms.

## Credit Author Statement

**Utkarsha Srivastava:** Investigation, Methodology, Data curation, Writing, Original draft preparation. **Ashwani Mathur:** Conceptualization, Visualization, Supervision, Draft revision.

## Data Availability

Mendeley DataData set on the GC–MS profile of Selaginella bryopteris from India (Original data).

## Footnotes

1 Codes used stand for the following: SBK- *S.bryopteris* from Karnataka, SBM- *S.bryopteris* from Maharashtra and SBT-*S.bryopteris* from Tamil Nadu.

2 Codes used in the text stand for the following: SBK-D is SBK extract in water, SBK-M is SBK extract in methanol, SBK-E is SBK extract in ethanol, SBK-A is SBK extract in acetone; SBM-D is SBM extract in water, SBM-M is SBM extract in methanol, SBM-E is SBM extract in ethanol, SBM-A is SBM extract in acetone; SBT-D is SBT extract in water, SBT-M is SBT extract in methanol, SBT-E is SBT extract in ethanol, SBT-A is SBT extract in acetone
